# Effect of phenylhexyl isothiocyanate on aberrant histone H3 methylation in primary human acute leukemia

**DOI:** 10.1186/1756-8722-5-36

**Published:** 2012-07-02

**Authors:** Yong Zou, Xudong Ma, Yiqun Huang, Lingling Hong, Jen-wei Chiao

**Affiliations:** 1 Department of Hematology, Zhangzhou Hospital of Fujian Medical University, Zhangzhou, Fujian Province, 363000, China; 2 Department of Medicine, New York Medical College, Valhalla, NY, 10595, USA

**Keywords:** Histone, Leukemia, Methylation, Phenylhexyl isothiocyanate (PHI)

## Abstract

**Background:**

We have previously studied the histone acetylation in primary human leukemia cells. However, histone H3 methylation in these cells has not been characterized.

**Methods:**

This study examined the methylation status at histone H3 lysine 4 (H3K4) and histone H3 lysine 9 (H3K9) in primary acute leukemia cells obtained from patients and compared with those in the non-leukemia and healthy cells. We further characterized the effect of phenylhexyl isothiocyanate (PHI), Trichostatin A (TSA), and 5-aza-2’-deoxycytidine (5-Aza) on the cells.

**Results:**

We found that methylation of histone H3K4 was virtually undetectable, while methylation at H3K9 was significantly higher in primary human leukemia cells. The histone H3K9 hypermethylation and histone H3K4 hypomethylation were observed in both myeloid and lymphoid leukemia cells. PHI was found to be able to normalize the methylation level in the primary leukemia cells. We further showed that PHI was able to enhance the methyltransferase activity of H3K4 and decrease the activity of H3K9 methyltransferase. 5-Aza had similar effect on H3K4, but minimal effect on H3K9, whereas TSA had no effect on H3K4 and H3K9 methyltransferases.

**Conclusions:**

This study revealed opposite methylation level of H3K4 and H3K9 in primary human leukemia cells and demonstrated for the first time that PHI has different effects on the methyltransferases for H3K4 and H3K9.

## Background

The N-terminal tails of histones go through a plethora of posttranslational modifications, including phosphorylation, ubiquitination, acetylation, and methylation. Each modification can affect chromatin architecture, and the sum of these modifications ultimately determines the chromatin state, which governs gene transcription [1,2]. Histone methylation has been implicated in multiple biological processes including heterochromatin formation, X-inactivation, genomic imprinting, and homeostatic gene silencing. Aberrant histone methylation has been linked to a number of human diseases such as cancer [3-7]. Methylation occurs on both lysine (K) and arginine residues. There are 5 lysine residues on the tails of histone H3 and one on H4 that are the focuses of research. These are histone H3 lysine 4(H3K4), H3K9, H3K27, H3K36, H3K79, and histone H4 lysine 20(H4K20). They reside within the core of histone proteins and have been shown to be the sites of methylation [8]. Methylation at these sites has been linked to both transcriptional activation and repression, as well as DNA damage response [9,10]. Methylation of H3K4 is associated with active chromatin in a wide range of eukaryotic organisms [11,12]. Methylation of H3K9 contributes to euchromatin gene silencing in many organisms [13]. Previous studies postulate that histone methylation is an irreversible process and can serve as a stable epigenetic mark. Enzymes that directly remove methyl marks from lysine and arginine residues of histones may provide a new level of plasticity within the system of epigenetic regulation [14-16].

Natural isothiocyanates (ITCs) are found in the cruciferous vegetables, genus *Brassica.* These include the common vegetables cabbages, broccoli, Boi choi (Chinese cabbage), etc. ITCs have been successfully synthesized. More than a dozen of ITC derivatives distinguish from the natural forms by the length in the aliphatic carbon chains. Phenylhexyl isothiocyanate (PHI) is one of the most potent ITC derivatives. Our group has shown that PHI suppressed growth of the leukemic cell line HL-60 by inducing apoptosis both in vitro and in vivo in immunodeficient mice. We further demonstrated that PHI has preferential activity in leukemia cells and spares normal cells and tissues [17,18]. PHI was also shown to suppress myeloma cell growth by inducing apoptosis and affecting VEGF production in vitro. PHI was also found to have effect on mitochondrial pathway [19]. PHI was found to cause epigenetic modifications both on histone and on DNA. PHI induced histone acetylation and DNA hypomethylation. We proposed for the first time that PHI has dual activity as a histone deacetylase inhibitor (HDACi) and as a DNA methyltransferase inhibitor [18,19]. We recently demonstrated aberrant histone acetylation in primary human leukemia cells and found that PHI can correct the abnormality in the cells [20,21].

In this report, we have identified the presence of aberrant histone methylation at H3K4 and H3K9 in fresh primary acute leukemia cells from patients. We further demonstrated that PHI is capable of correcting the aberrant methylation in the human leukemia cells. We demonstrated for the first time that PHI has differential effects on methyltransferases for H3K4 and H3K9.

## Patients and methods

### Study subjects

The laboratory analysis of bone marrow and peripheral blood specimens after diagnosis were approved by the human ethics committee of Zhangzhou Affiliated Hospital of Fujian Medical University, China. Informed consent was obtained. This study analyzed samples from twenty seven patients with primary untreated acute leukemias. These patients were enrolled from September 2007 to December 2008, including patients with ALL (acute lymphoblastic leukemia), AML (acute myeloid leukemia), or BAL (biphenotypic acute leukemia) with their age ranged from 6–78.

Histone methylation was examined in the mononuclear cells isolated from the bone marrow of nineteen patients by Ficoll-Hypaque density gradient centrifugation. These patients included eleven males and eight females, with a median age of 38 (6–78). They included one case of M1, two cases of M2a, three cases of M3a, one case of M5a, two cases of M5b, three cases of L1, five cases of L2 and two cases of BAL. To study the effect of PHI on leukemia cells, bone marrow mononuclear cells were isolated from an additional group of eight patients. The cells were cultured and treated with PHI, tricostatin (TSA) and 5-Aza-2-dexoy-citidine (5-Aza). These patients included five males and three females, with a median age of 34 (12–78). They included two cases of M2a, one case of M3a, one case of M5b, one case of L1, two cases of L2 and one case of BAL. Individuals without leukemia, including two patients with iron-deficiency anemia (IDA), one with infection disease, two with idiopathic thrombocytopenic purpura (ITP), as well as three healthy volunteers were enrolled in the study and served as controls.

### Cell cultures

PHI, greater than 98% pure, was purchased from LKT Lab (St. Paul, MN). PHI was dissolved in a stock solution of 75% methanol. TSA (Sigma Inc.) was dissolved in dimethyl sulfoxide (DMSO). 5-Aza (Sigma Inc) was prepared in phosphate buffer (PBS). Bone marrow mononuclear cells, isolated from eight acute leukemia (AL) patients (P20-P27, Table 1) containing 56-92% blasts, and HL-60 cell line (from Shanghai typical cell culture center), were seeded at 5 x 10^5^ cells/ml of RPMI-1640 medium with 15% heat-inactivated fetal calf serum, 100 IU/ml of penicillin and streptomycin, and maintained at 37 °C in a 5% CO_2_ atmosphere. Cultures were exposed to various concentrations of PHI (0, 10, 20, 40 μmol/L), TSA at 10 μmol/L, or 5-Aza at 10 μmol/L. Cultures supplemented with the methanol medium were used as a vehicle control. After PHI treatment, bone marrow cell preparations were evaluated for histone methylation at H3K4 and H3K9 by Western blotting.

**Table 1:** Characteristics of leukemia patients

**patient**	**Age(yr)**	**sex**	**sample**	**diagnosis**	**blast(%)**	**Treatment**	**response**	**culture**
P1	50	M	BM	M1	90	MA	no	no
P2	41	M	BM	M2a	62	MA	CR	no
P3	40	F	BM	M2a	81	MA	CR	no
P4	27	F	BM	M3a	92	ATRA + AA	CR	no
P5	27	M	BM	M3a	76	ATRA + AA	CR	no
P6	34	M	BM	M3a	68	ATRA + AA	CR	no
P7	16	F	BM	M5a	93	MA	CR	no
P8	78	F	BM	M5b	70	MA	PR	no
P9	12	M	BM	M5b	60	MA	CR	no
P10	6	M	BM	L1	92	VMLP	PR	no
P11	48	F	BM	L1	95	VMLP	PR	no
P12	48	M	BM	L1	83	VMLP	CR	no
P13	17	M	BM	L2	76	VMLP	CR	no
P14	46	F	BM	L2	85	VMLP	CR	no
P15	66	M	BM	L2	92	VMLP	PR	no
P16	66	F	BM	L2	90	VMLP	CR	no
P17	36	M	BM	L2	88	VMLP	CR	no
P18	42	F	BM	BAL	92	MA + VP	PR	no
P19	22	M	BM	BAL	80	MA + VP	PR	no
P20	50	M	BM	M2a	56	MA	PR	yes
P21	41	M	BM	M2a	92	MA	PR	yes
P22	10	F	BM	M3a	90	ATRA + AA	CR	yes
P23	12	F	BM	M5b	90	MA	CR	yes
P24	26	M	BM	L1	76	VMLP	PR	yes
P25	16	M	BM	L2	80	VMLP	CR	yes
P26	39	M	BM	L2	82	VMLP	CR	yes
P27	78	F	BM	BAL	62.4	MA + VP	PR	yes

Abbreviations: M = male; F = female; BM = bone marrow; CR = complete remission; PR = partial remission. AML=acute myeloid leukemia; ALL=acute lymphoblastic leukemia; BAL= biphenotypic acute leukemia MA = mitoxantrone, cytarabine; VMLP = vincristine, mitoxantrone, asparaginase, prednisone; ATRA + AA = all-trans-retinoic acid + arsenic trioxide; VP = vincristine, prednisone; M1-M5b and L1-L2 are the FAB classifications for AML and ALL.

### Evaluation of histone methylation

Bone marrow mononuclear cells from 19 leukemia patients (P1-P19, Table 1) were isolated by Ficoll-Hypaque density gradient centrifugation. The preparations that contained 90% or more blasts by morphology were used for analysis. Bone marrow mononuclear cells from five non-leukemia patients and the peripheral blood mononuclear cells from three healthy volunteers were isolated as described above. Lysates of the mononuclear cells were prepared with a Pierce lysis buffer (Pierce, Rockford, IL) and stored at −80 °C. Western blotting was performed according to previously described methods. Proteins were separated by electrophoresis and immunoblotted with specific anti-mono-methylated histone H3K4 or H3K9 antibodies (Upstate Biochenology, Lake Placid, NY). β-actin was used as a loading control. To quantify the protein expression level, the protein density from Western blotting was determined by a color image analysis system (AlphaDigiDoc, Alpha Innotech).

### Quantification of histone methyltransferase activity

To compare the histone H3 methyltransferase activity, HL-60 cells were cultured as described above for cell culture. EpiQuik Histone Methyltransferase Activity/Inhibition Assay kits (Epigentek Group Inc, NY) specific for H3K4 and H3K9 modifications were used to quantify the H3 methyltransferase activity. The procedure was done according to the manufacturer’s instructions (Epigentek). For methylation assays, a blank lane was always included as a control to determine the spontaneous background.

### Statistical analysis

Statistical analysis was performed with the software SPSS 11.5. *P*≤0.05 was considered to be statistically significant.

## Results

### Aberrant histone methylation in primary human leukemia cells

**Figure 1:**
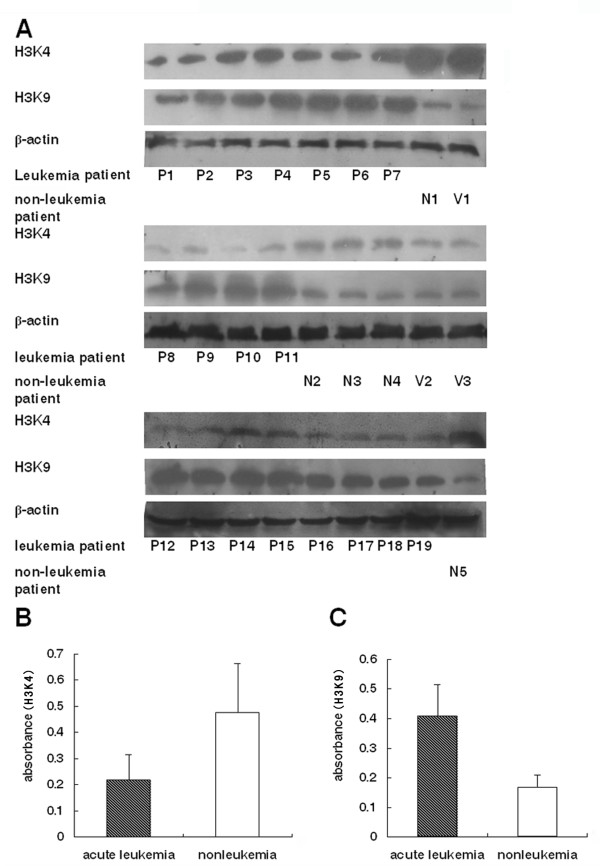
**Histone H3 methylation in primary human leukemia cells.** The methylation status of histone H3 lysine 4 (H3K4) and H3 lysine 9 (H3K9) was examined by Western blotting. Panel A, top group, shows the expression level of methylated H3K4 and H3K9 in primary marrow mononuclear cells isolated from seven acute leukemia cases (P1-P7), one individual without leukemia (N1), and one healthy volunteer (V1). The middle and lower groups of panel A depict the level of methylated H3K4 and H3K9 in the cells from additional twelve acute leukemia cases (P8-P19), non-leukemia individual with IDA (N2), one with infection (N3), two with ITP (N4, N5), and two healthy volunteers (V2, V3). β-actin was used as a loading control. The expression level of the methylated H3K4 (Panel B) and H3K9 (panel C) in primary leukemia cells from all leukemia patients (P1-P19) was compared with those of non-leukemia cells (N1-N5 and V1-V3). The vertical bars in panels B and C represent the mean ± SD of the densities of methylated H3K4 and H3K9, quantified as densitometric unit value in absorbance from the Western blottings in panel A. (▓) indicates the level of methylated H3K4 (panel B) and H3K9 (panel C) from the 19 leukemia patients (P1-P19), and (□) indicates the level of methylated H3K4 and H3K9 from the 8 non-leukemia subjects (N1-N5, V1-V3). Panel B shows that the histone H3K4 in acute leukemia cells is significantly lower than that in the non-leukemia cells (*P* < 0.05). Panel C shows that the histone H3K9 in acute leukemia cells is significantly higher than that in the non-leukemia cells (*P* < 0.05).

The methylation status of histone H3 at lysines 4 and 9 was compared in mononuclear cells from patients with acute leukemia, and from individuals without leukemia and health volunteers (Figure 1). The level of mono-methylated histone H3K4 and H3K9 from seven AL patients (P1-P7), one healthy volunteer (V1), and one anemia patient (N1) was characterized by Western blotting (top panel of Figure 1A). The methylation level at histone H3K4 in each leukemia patient was significantly lower, while methylation at H3K9 was higher, than that in the non-leukemia and healthy cells. This observation was confirmed in additional twelve patients with acute leukemia (P8-P19), as revealed in the middle and lower panels (Figure 1A). The non-leukemia specimens were from one patient with iron-deficient anemia (N2), three with ITP (N3, N4, N5), and two healthy donors (V2, V3). As depicted in Figure 1B, the methylation level at H3K4 in leukemia cells (0.2199 ± 0.096) is less than half of that in the non-leukemia cells (0.4773 ± 0.186). The difference is statistically significant (*P* = 0.005). Figure 1C shows that the methylation level at H3K9 (0.4089 ± 0.106) in leukemia cells is more than 2 X higher than that in the non-leukemia controls (0.1675 ± 0.0414), with the difference statistically significant (*P* = 0.001). These results clearly demonstrated that histone H3 was hypomethylated at H3K4, and hypermethylated at H3K9 in leukemia patients.

### Effect of PHI on histone H3 methylation

**Figure 2:**
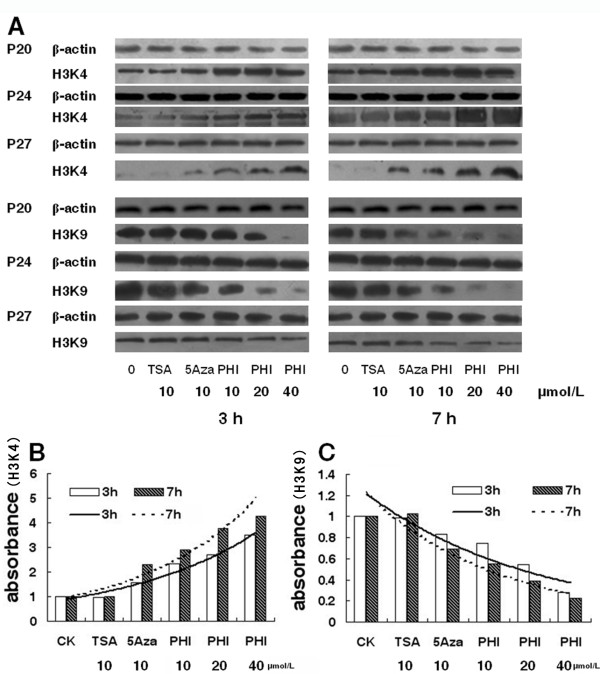
**The effect of phenylhexyl isothiocyanate, trichostatin (TSA) and 5-aza-2-deoxycitidine (5-Aza) on histone H3 methylation in primary human leukemia cells.** The methylation status of histone H3 lysine 4 (H3K4) and H3 lysine 9 (H3K9) was examined by Western blotting. Panel A shows a dose- and time-related reduction of methylation at histone H3K4 (top group) and H3K9 (lower group) in three patients (P20, P24, P27) after exposure for 3 and 7 hours, respectively, to PHI, TSA, and 5-Aza. The level of methylated H3K4 (panel B) and H3K9 (panel C) of eight patients was quantitated by densitometry. β-actin was used as a loading control (CK). The highest level was marked by the lines.

We have demonstrated that PHI enhanced acetylation of histones H3 and H4 [18,20,21]. In this study we examined the methylation of histone H3 in primary human acute leukemia cells from eight patients (P20-P27 Table 1), We put three cases of them on the Figure 2A(including P20, P24, P27), PHI altered the level of histone methylation in a dose- and time-dependent manner (Figure 2). After a three-hour exposure to PHI, the level of mono-methylated histone H3K4 was significantly increased, leading to normalization of methylation in the primary leukemia cells. The level was 1.71 times higher in cells treated with 20 μmol/L of PHI, and 2.48 times higher with 40 μmol/L after three hours of exposure (Figure 2 B). After a seven-hour exposure, the methylation level of H3K4 was further increased, 2.77 times higher than that of the control when cells were treated with 20 μmol/L PHI, and 3.28 times higher with 40 μmol/L. On the other hand, methylation level at H3K9 in the patients’ cells was significantly attenuated after PHI treatment. The mono-methylation was reduced by 46% in the leukemia cells treated with 20 μmol/L PHI, and 73% with 40 μmol/L PHI after a three-hour exposure, when compared to that in the control cells. After a seven-hour exposure, the methylation level was further reduced by 61% with 20 μmol/L PHI and 77% with 40 μmol/L PHI (Figure 2 C). In the meantime, TSA used at 10 μmol/L had no effect on the methylation level at both histone H3K4 and H3K9. 5-Aza at 10 μmol/L revealed a minor effect on methylation at H3K4 and H3K9 (Figure 2B and 2C).

###  PHI activated H3K4 histone methyltransferase and inhibited H3K9 methyltransferase

**Figure 3:**
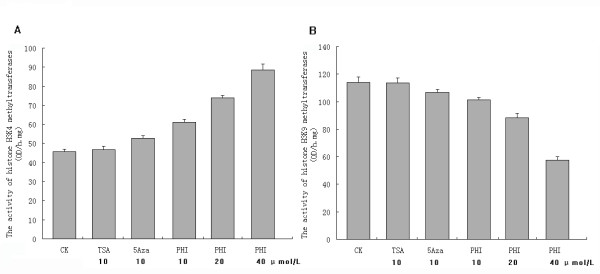
**Effect of phenylhexyl isothiocyanate (PHI), trichostatin (TSA) and 5-aza-2-deoxycitidine (5-Aza) on histone H3 lysine 4 and lysine 9 methyltransferases in HL-60 human leukemia cell line.** The methyltransferase activity for histone H3 lysine 4 (H3K4, panel A) and H3 lysine 9 (H3K9, panel B) in HL-60 cells was measured by colorimetric methylation assay as described in the Methods. The bar graph represents the results from five independent assays. β-actin was used as a loading control (CK).

To further study the mechanism, we examined the methytransferase activity in HL-60 cell line. Figure 3A showed that PHI activated histone methyltransferase for H3K4. Activity of H3K4 methyltransferase was 60.97 ± 1.75, 73.97 ± 1.21 and 88.52 ± 3.16 O.D/h/mg, after exposure to PHI at 10, 20, and 40 μmol/L for 7 hours. Compared to the control (45.58 ± 1.53 O.D./h/mg), the level of enzyme activity is significantly higher (p < 0.01). 5-Aza also activated the H3K4 methyltransferase significantly (52.66 ± 1.39 O.D./h/mg) at 10 μmol/L (p < 0.05). However, it was significantly less effective than PHI at the same concentration (p < 0.05). TSA had no effect on H3K4 methyltransferase (46.80 ± 1.84 O.D./h/mg, p = 1.00). Figure 3B showed that PHI inhibited H3K9 methyl-transferases. The activity of the histone methyltransferase on H3K9 was 101.12 ± 2.11, 88.13 ± 3.25 and 57.58 ± 2.42 O.D./h/mg after exposure to PHI at 10, 20 and 40 μmol/L for 7 hours. Compared to that in the control cells (114.12 ± 3.69 O.D./h/mg), the activity was significantly lower (p < 0.01). However, 5-Aza and TSA had no significant effect on H3K9 methyltransferase (5-Aza, 106.68 ± 2.31 O.D./h/mg, p = 0.09; and TSA, 113.74 ± 3.39 O.D./h/mg, p = 0.87) (Figure 3B).

## Discussion

This study demonstrated for the first time that PHI has different effects on the methyltransferases of H3K4 and H3K9 in the leukemia cells. The methylation at H3K4 is generally associated with transcriptional activation, while methylation of H3K9 signifies heterochromatinization and transcriptional inhibition of euchromatin [22,23]. Our findings suggest the possible presence of an aberrant chromatin structure in acute leukemia cells. The differential levels of methylation status at H3K4 and H3K9 could potentially serve as a novel biomarker for the acute leukemia.

Methylation and acetylation of histone H3 are interconnected post-translational modifications. The methylation of histone H3K4 appears to correlate with its acetylation. PHI is known to augment acetylation at H3K4 via augmenting the activity of P300 [18,20], which might promote methylation since acetylated isoforms of H3 are known as the preferential targets of histone methylases [24,25]. SET1, SET7/9, Ash1, ALL-1, MLL, ALR, Trx and SMYD3 are contained in histone methyltransferases that catalyze methylation of H3K4 in mammalian cells [26-28]. ESET, G9a, SUV39-H1, SUV39-H2, SETDB1, Dim-5 and Eu-HMTase are histone methyltransferases that catalyze methy-lation of H3K9 in mammalian cells [29-32]. It appears that 5-Aza activates H3K4 histone methyltransferase but has minimal effect on H3K9 methyltransferase. TSA has negligible effect on either histone methyltransferases. This intriguing finding suggests that PHI may be a unique and novel agent that regulates histone H3 methyltransferases, different from those of 5-azacitidine and decitabine [33]. It would be interesting to explore whether PHI could be among many new agents being studied for novel cancer therapy [16,34-36]. It is also important to study the combination effects of PHI/ITCs with different classes of agents with novel mechanisms of action [37,38].

## Abbreviations

H3K4, Histone H3 lysine4; H3K9, Histone H3 lysine9; K, Lysine; PHI, Phenyhexyl isothiocyanate; TSA, Trichostatin A; 5-Aza, 5-aza-2’-deoxycytidine; IDA, Iron deficiency anemia; ITP, Idiopathic thrombocytopenic purpura; PBS, Phosphate buffer; AML, Acute myeloid leukemia; ALL, Acute lymphoblastic leukemia; AL, Acute leukemia; BAL, Biphenotypic acute leukemia; HDACi, Histone deacetylase inhibitor; ITC, Isothiocyanate; DMSO, Dimethyl sulfoxide.

## Competing interests

The authors declare that they have no competing interests.

## Authors’ contributions

XM is responsible for study design, data analysis, and manuscript preparation. YZ and YH carried out laboratory study and data collection. LH is responsible for collecting data and analyzing the data. JwC is involved in manuscript preparation. All authors have read and approved the final manuscript.
